# Sex Differences of Human Cardiac Progenitor Cells in the Biological Response to TNF-*α* Treatment

**DOI:** 10.1155/2017/4790563

**Published:** 2017-09-17

**Authors:** Elisabetta Straface, Lucrezia Gambardella, Francesca Pagano, Francesco Angelini, Barbara Ascione, Rosa Vona, Elena De Falco, Elena Cavarretta, Raffaele La Russa, Walter Malorni, Giacomo Frati, Isotta Chimenti

**Affiliations:** ^1^Center for Gender-Specific Medicine, Istituto Superiore di Sanità, Rome, Italy; ^2^Department of Medical Surgical Sciences and Biotechnologies, “La Sapienza” University of Rome, Rome, Italy; ^3^Department of Anatomical, Histological, Forensic and Orthopaedic Sciences, Sapienza University of Rome, Viale Regina Elena 336, 00185 Rome, Italy; ^4^Malzoni Clinical-Scientific Institute (MaCSI), Via Carmelo Errico 2, 83100 Avellino, Italy; ^5^Department of AngioCardioNeurology, IRCCS Neuromed, Pozzilli, Italy

## Abstract

Adult cardiac progenitor cells (CPCs), isolated as cardiosphere-derived cells (CDCs), represent promising candidates for cardiac regenerative therapy. CDCs can be expanded in vitro manyfolds without losing their differentiation potential, reaching numbers that are appropriate for clinical applications. Since mechanisms of successful CDC survival and engraftment in the damaged myocardium are still critical and unresolved issues, we aimed at deciphering possible key factors capable of bolstering CDC function. In particular, the response and the phenotype of CDCs exposed to low concentrations of the multifunctional cytokine tumor necrosis factor *α* (TNF-*α*), known to be capable of activating cell survival pathways, have been investigated. Furthermore, differential biological responses of CDCs from male and female donors, in terms of cell cycle progression and cell spreading, have also been assessed. The results obtained indicate that (i) the intracellular signaling activated in our experimental conditions is most likely due to the prosurvival and proliferative signaling of TNF-*α* receptor 2 and that (ii) cells from female patients appear more responsive to TNF-*α* treatment in terms of cell cycle progression and migration ability. In conclusion, the present report highlights the hypothesis that TNF-stimulated CDCs isolated from females may represent a promising candidate for cardiac regenerative therapy applications.

## 1. Introduction

Cardiovascular diseases remain the leading cause for morbidity/mortality in the Western world [1, 2]. Translational research and recent clinical trials suggest that adult stem/progenitor cell (S/PC) transplantation into the damaged myocardium (cell therapy) can improve cardiac function [3, 4]. To regenerate the heart and restore its function, many types of S/PCs are currently being explored, each with their own benefits and limitations. Cardiac cell therapy with S/PCs from extracardiac tissues (e.g., mesenchymal stem cells and bone marrow mononuclear cells) can decrease the death rate of endogenous myocytes and improve neoangiogenesis, probably by secretion of paracrine factors, such as several cytokines, chemokines, and growth factors [5]. These factors have been shown to reduce inflammation, decrease apoptotic cell death, and improve overall myocardial function [6]. Direct regeneration has been shown to be more efficient with the use of resident cardiac progenitor cells (CPCs) [7], which nonetheless exert paracrine effects [6, 8]. Adult CPCs are multipotent cells isolated from heart tissue obtained from patients undergoing surgery or catheterization and can be isolated as spontaneous spheroids, named cardiospheres (CSs) [9]. These cells can be expanded in vitro manyfolds without losing their differentiation potential, reaching numbers that are appropriate for in vivo transplantation in patients [10]. CSs can be efficiently isolated even from advanced heart failure patients [11], and the only medical parameter described so far to significantly impair CS isolation efficiency and affect CS phenotype is beta-blocker assumption by tissue donors [12]. Despite promising preclinical and clinical results, several limitations still exist for cardiac cell therapy. In particular, the need to improve survival, differentiation, and engraftment of the transplanted stem cell population [13], even with resident CPCs [14], has been envisaged. Thus, the first step to optimize the repair of a damaged myocardium by transplanted S/PCs is to increase their survival. Insufficient homing and engraftment of transplanted cells into the ischemic milieu limit in fact the full potential of cell-based cardiac repair, and several approaches have been introduced to overcome these limitations, such as tissue engineering [15] or pharmacological pre-/postconditioning [16, 17].

Multiple molecules have been described to mediate the complex and hostile signals in diseased cardiac tissue during injury and remodeling. Among these, tumor necrosis factor alpha (TNF-*α*) seems to play a critical role. This is a potent multifunctional cytokine involved in a number of pathologies in the cardiovascular system, including heart failure, and associated with many diverse physiological and physiopathological opposite events, such as cell death by apoptosis, but also cell growth, differentiation, and cell survival depending on the dose [18]. In line with this, a dual role of TNF-*α* in the attenuation or in the aggravation of cardiac injury has been proposed. Paradoxically, it has in fact been reported that low levels of TNF-*α* can trigger a cardioprotective program via the well-described free radical signaling pathway [19], whereas high concentrations of this cytokine are responsible of its well-known proapoptotic and inflammatory cascades [20]. Concerning CS biology, multiple signaling pathways have been reported so far to affect their phenotype, including those related to thrombin [21], EGF [22], IGF1 and Wnt [23, 24], TGF-beta [25], and beta-adrenergic signaling [12, 26]. However, the influence of TNF-*α* on the biology of CPCs has not been thoroughly investigated yet.

Since abundant data from the literature underscore a significant disparity between men and women for the incidence and severity of cardiovascular diseases [27], several investigations aimed at elucidating the mechanisms underlying these differences have been carried out. It has been observed that men undergo more rapid progression of heart failure, less preservation of myocardial mass as they age, and worse age-matched cardiac contractility compared to women. However, the possible implication of CPCs in this disparity has been poorly investigated. For instance, sex-related differences in the abundance of resident CPCs have never been detected so far [11, 12, 28], and sex-related differences in the response to inflammatory signals have not been investigated yet. However, interestingly, a differential cardioprotective role of TNF-*α* has been described in mesenchymal stem cell (MSC) therapy based on the sex of MSC donors. In fact, MSCs from males seem to be more sensitive to TNF-*α*-mediated detrimental signals, that is, at high concentrations of the cytokine, than their female counterparts [29]. On the basis of these considerations, CPCs in the form of CS-derived cells from male and female donors have been exposed to low, noncytotoxic concentrations of TNF-*α* in order to investigate their differential biological response.

## 2. Materials and Methods

### 2.1. Cardiosphere and Cardiosphere-Derived Cell Isolation, Culture, and Treatments

Human auricola biopsies were obtained from nine patients (five males and four females) undergoing cardiac surgery for ischaemic cardiomyopathy, after informed consent and under protocol number 2154/15, approved by the Ethical Committee of the “Umberto I” Hospital, “La Sapienza” University of Rome. Available anthropometric and medical data are reported in Table 1. Donors were all under beta-blocker treatment [12]. CPCs were isolated with the standardized CS protocol, as previously described [30]. Briefly, after approximately 4 weeks of explant outgrowth on fibronectin-coated Petri dishes (BD Biosciences) in complete explant medium (CEM; IMDM medium (Gibco) supplemented with 1% penicillin-streptomycin, 1% L-glutamine, 0.1 mM 2-mercaptoethanol (Gibco), and 20% FBS (Lonza)), explant-derived cells (EDCs) were collected every 7 days (up to 3 times from each explant) and seeded on poly-D-lysine- (BD Biosciences) coated wells (7000 cells/cm^2^) to obtain CSs in CS-growth medium (CGM): 35% IMDM/65% DMEM/F-12 Mix (Gibco and Lonza), 3.5% FBS, 1% penicillin-streptomycin, 1% L-glutamine, 0.1 mM 2-mercaptoethanol, 1 unit/ml thrombin (Sigma-Aldrich), 1 : 50 B-27 (Invitrogen), 80 ng/ml bFGF, 25 ng/ml EGF (Peprotech), and 4 ng/ml cardiotrophin-1 (Peprotech). After 1 week, CSs were collected and expanded on fibronectin-coated surfaces in CEM as cardiosphere-derived cells (CDCs) and expanded for not more than three split rounds. Cells were treated with 100 ng/ml TNF-*α* (Sigma-Aldrich) for up to 48 hours.

### 2.2. Expression of TNF-*α* Receptors

To verify the expression of TNF-*α* receptor 1 (TNFR1) and TNF-*α* receptor 2 (TNFR2) on the cell surface, cells were incubated with R-phycoerythrin-conjugated murine antibodies against human TNFR1 and TNFR2 (Caltag Laboratories, Burlingame, CA, USA). Samples were analyzed on a FACScan flow cytometer by using the Cell Quest software (Becton Dickinson, Mountain View, CA, USA).

### 2.3. Evaluation of the Redox State

Cells (5 × 10^5^) were incubated with 1 *μ*mol/l of dihydroethidium (DHE, Molecular Probes) or 10 *μ*mol/l of dihydrorhodamine 123 (DHR 123, Molecular Probes) for 15 minutes at 37°C. After washing, samples were analyzed on a FACScan flow cytometer by using the Cell Quest software (Becton Dickinson, Mountain View, CA, USA).

### 2.4. Evaluation of Apoptosis

Quantitative evaluation of apoptosis was performed by flow cytometry after double staining using fluorescein isothiocyanate-conjugated annexin V and 0.05% trypan blue for 10 minutes at room temperature and analyzed by flow cytometry in the FL1 and FL3 channels to determine the percentage of dead cells [31]. Samples were analyzed on a FACScan flow cytometer by using the Cell Quest software (Becton Dickinson, Mountain View, CA, USA).

### 2.5. Cell Cycle

Cell cycle analyses were conducted after 48 hours of TNF-*α* treatment. Cultured cells were treated with 1 mmol/l bromodeoxyuridine (BrdU; BD Immunocytometry Systems) for 30 minutes and fixed in 70% ice-cold ethanol. 1 × 10^6^ fixed cells were incubated in 3 N HCl for 20 minutes. After washing with 0.1 mol/l Na_2_B_4_O_7_ (pH 8.5) to stop acid denaturation, cells were washed twice with 1% bovine serum albumin and 0.5% Tween-20 and then labeled with an anti-BrdU FITC-conjugated antibody (BD Immunocytometry Systems) for 30 minutes at 4°C. Cells were then stained with 40 *μ*g/ml PI (Sigma-Aldrich) in the presence of 10 *μ*mol/l RNase (Sigma-Aldrich) for 30 minutes at 37°C. Sample analysis was performed on a FACScan flow cytometer by using the Cell Quest software (Becton Dickinson, Mountain View, CA, USA).

### 2.6. Analytical Cytology

For nuclear factor kappa B (NF-*κ*B) detection, cells were fixed in acetone/methanol 1/1 (*v/v*) for 10 minutes at room temperature and air dried. After 1 hour of preincubation with PBS containing 10% of AB human serum, cells were incubated for 1 hour at room temperature with the rabbit polyclonal antibody to NF-*κ*B (Santa Cruz Biotechnology). Following three washes in PBS, cells were incubated for 1 hour at room temperature with FITC-labeled anti-rabbit secondary antibody. Morphometric analyses were also employed to evaluate NF-*κ*B nuclear translocation. The cells with positive nucleus were evaluated by counting 300 cells at high magnification (500x). The nuclei were stained with Hoechst 33258 (Sigma-Aldrich) at 37°C for 15 minutes. For actin filament detection, cells were fixed with 4% paraformaldehyde, permeabilized with 0.5% Triton X-100 (Sigma-Aldrich), and stained with fluorescein-conjugated phalloidin (Sigma-Aldrich) at 37°C for 30 minutes.

All samples were mounted on glass cover slips with glycerol/PBS (2 : 1) and observed by intensified video microscopy (IVM) with an Olympus Microphot fluorescence microscope (Olympus Corporation, Tokyo, Japan) equipped with a Zeiss CCD camera.

### 2.7. Scratch Assay

Cell migration was examined by scratch assay according to Liang et al. [32]. Approximately 2.5 × 10^5^ cells were seeded in 35 mm Petri dishes. When cells reached confluence, dishes were scratched with a sterile 200 *μ*l pipette tip, treated or not with TNF-*α*, and incubated at 37°C.

Migration of cells towards wound closure of the same region at 0 and 24 hours was monitored, and images were acquired using a digital camera system coupled with an inverted microscope (Olympus IX-71). Repopulation by migrating cells of the wound region was then analyzed and quantified using the ImageJ v1.48 software (http://imagej.nih.gov/ij/).

### 2.8. Data Analysis and Statistics

Cytofluorimetric results were statistically analyzed by using the parametric Kolmogorov–Smirnov test using Cell Quest software. A least 20,000 events were acquired. The median values of fluorescence intensity histograms were used to provide a semiquantitative analysis. The results are displayed as average value ± standard deviation, unless otherwise specified. Significance of difference between any two groups was assessed by two-sided Student's *t-*test. A threshold value of *p* < 0.05 was considered to be significant.

## 3. Results and Discussion

### 3.1. Antiapoptotic Response of Cardiac Progenitor Cells to TNF-*α*

In order to rule out any significant baseline difference in the differentiation potential of male versus female CDCs, we performed a preliminary real-time PCR gene expression screening for a panel of markers of cardiovascular commitment, stemness, and epithelial-to-mesenchymal transition. No statistically significant differences could be detected between CDCs from males and females (Supplementary Figure 1A available online at https://doi.org/10.1155/2017/4790563).

**Figure 1:**
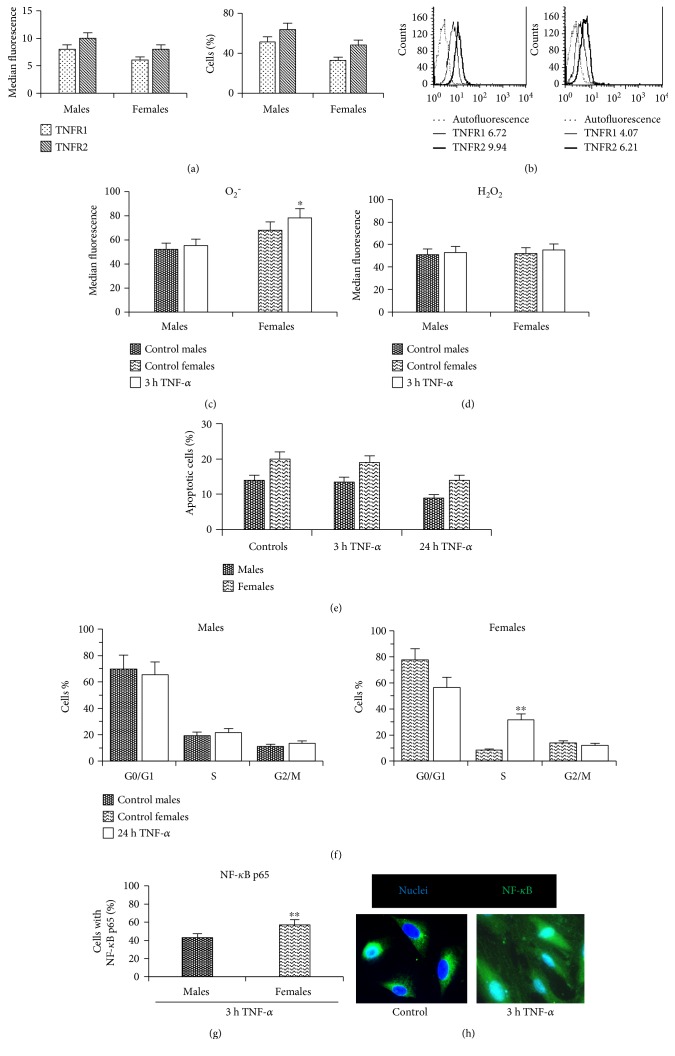
Antiapoptotic effect of TNF-*α* in CPCs. Cytometric analysis of (a) TNFR1 and TNFR2 mean fluorescence intensity (left histogram) and percentage of positive cells (right histogram), (b) representative flow cytometry histograms showing TNFR expression in cells from a male and from a female patient, (c) superoxide anion (O_2_^−^), and (d) hydrogen peroxide (H_2_O_2_) levels; (e) percentage of apoptotic cells after 3 and 24 hours of TNF-*α* treatment. (f) Cell cycle analysis after propidium iodide staining. Data are reported as mean values ± SD of three independent experiments. (g) Morphometric analysis showing the percentage of cells with NF-*κ*B p65 3 h after TNF-*α* treatment. (h) Two representative images obtained by fluorescence microscopy of female control cells with cytoplasmic NF-*κ*B and female cells after 3 h of TNF-*α* treatment with NF-*κ*B p65 localization. ^∗^*p* < 0.05. ^∗∗^*p* < 0.001.

Then, we investigated possible gender-related differences in TNF-*α* responsiveness. The effects of TNF-*α* are mediated by two receptors called TNFR1 and TNFR2. To evaluate the expression of TNF receptors in male/female CDCs, flow cytometry analysis was performed. Interestingly, we found that both TNFR1 and TNFR2 are expressed by CDCs from male and female patients (Figure 1(a), left histogram), albeit in a lower percentage of female cells (Figure 1(a) right histogram) and/or at a global lower expression level, as suggested by the homogeneous peak shift in the representative histogram panel (Figure 1(b)). TNFR1 activation generates reactive oxygen species (ROS) and induces apoptosis [33]. Conversely, TNFR2, although generating ROS-mediated signaling pathways, does not contain a death domain and cannot transmit proapoptotic signals. Its activation leads to cell survival, proliferation, and growth factor production [29]. TNFR1 is expressed ubiquitously on almost all cell types. TNFR2 expression, instead, is restricted to certain cell types including endothelial cells, myocytes, thymocytes, and human mesenchymal stem cells. Recently, published data have revealed that these two receptors not only function independently but can also influence each other via cross talk between the different signaling pathways initiated by TNFR1 and TNFR2 stimulation [34].

Next, we investigated the effects of TNF-*α* treatment. CDCs have been reported to release low levels of TNF-*α* in culture [8], but the in vitro concentration of 100 ng/ml used in the present study far exceeds the amount due to autocrine production. Considering that ROS appear to serve as key mediators involved in TNF-*α*-induced cellular responses [19], superoxide anion (O_2_^−^) and hydrogen peroxide (H_2_O_2_) levels were measured by flow cytometry at different time points (3, 6, and 24 hours) after TNF-*α* treatment. After 3 hours of TNF-*α* treatment, a higher increase of O_2_^−^ levels was detectable in stimulated female versus male cells, compared to control (Δ = 6% in males and Δ = 15% in females, *p* < 0.05 versus control), while H_2_O_2_ levels were comparable (Δ = 4% in cells from males and Δ = 6% in cells from females) (Figures 1(c) and 1(d)). Gene expression levels for different NADPH oxidase isoforms, which are the main ROS-producing enzymes [35, 36], were comparable between male and female CDCs (Supplementary Figure 1B), thus excluding any baseline difference between the two.

To determine whether ROS production after TNF-*α* treatment of CDCs exerted an apoptotic or antiapoptotic cell response, apoptosis and cell cycle progression were assessed by flow cytometry. We found that 24 hours after TNF-*α* treatment, no difference in the percentage of apoptotic cells was detected in both cell lines (Figure 1(e)). Conversely, a significant (*p* < 0.001) increase in the percentage of cells in S phase of the cell cycle compared to untreated controls was detected in CDCs from females 48 hours after TNF-*α* treatment whereas no significant changes were observed in CDCs from males after the same treatment protocol (21.0 ± 0.4% versus 19.0 ± 0.5%) (Figure 1(f)). On the basis of these data, we hypothesized that TNF-*α*, at the concentration here considered, did not exert a proapoptotic effect in CDCs from both male and female patients, suggesting that TNF-*α* signaling through TNFR2 could be dominant.

This hypothesis was further supported by the analysis of nuclear localization of nuclear factor kappa B transcription factor subunit p65 (NF-*κ*B p65). In fact, nuclear localization is a ROS-dependent process of cell activation that was observable in cells from both male and female patients 3 hours after TNF-*α* treatment. At this time, NF-*κ*B p65 subunit was found in 44.0 ± 1.9% of cells from males and in 60.0 ± 1.5% of cells from females, with a statistically significant difference between them (Figure 1(g)).  Figure 1 shows two representative images obtained by fluorescence microscopy, indicating control cells with cytoplasmic labeling of NF-*κ*B p65 subunit and cells after 3 hours of TNF-*α* treatment characterized by nuclear positivity. Interestingly, we found that the percentage of cells from female patients with NF-*κ*B p65 subunit was significantly (*p* = 0.001) higher than that obtained from male patients. This was in line with the literature suggesting that, in the absence of NF-*κ*B activity, cellular susceptibility to TNF-induced apoptosis increases, whereas enforced activation of NF-*κ*B is able to protect cells from apoptosis [37]. Overall, the results showed that (1) both TNFR1 and TNFR2 were expressed by CDCs from both male and female patients, (2) low levels of ROS were generated by TNF-*α* treatment in CDCs from both male and female patients, (3) no differences in apoptosis were detected after 3 and 24 hours of TNF-*α* treatment in cells of both genders, (4) a significant (*p* < 0.001) percentage of cells in S phase was detected after TNF-*α* in CDCs from female patients with respect to those from males, and (5) a significant (*p* = 0.001) increase of cells with NF-*κ*B was detected in cells from females with respect to those from male patients 3 hours after TNF-*α*.

### 3.2. Effect of TNF-*α* on Actin Filament Organization and Migration

**Figure 2:**
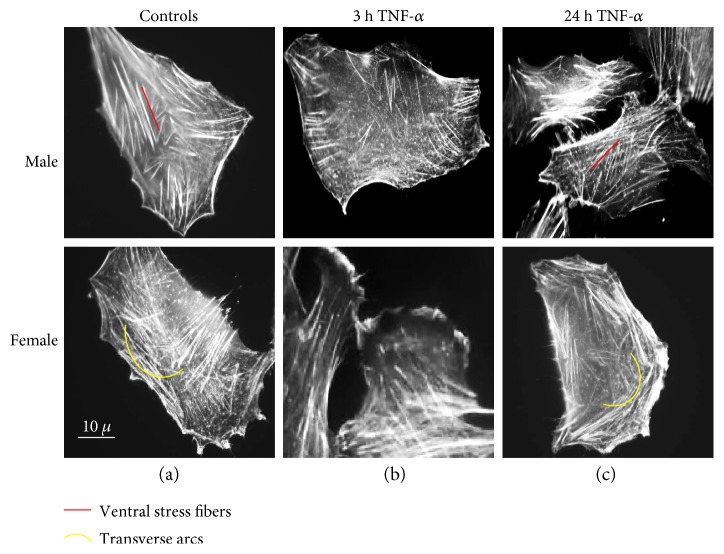
Effect of TNF-*α* on actin filament organization. Static cytometry analysis of actin cytoskeleton in cells stained with fluorescein—phalloidin. Numerous ventral stress fibers in control cells from males and transverse arcs in control cells from females (a) are detectable; stress fiber loss and intense lamellipodia formation are visible 3 hours after TNF-*α* treatment (b) and reappearance of stress fibers after 24 hours of TNF-*α* treatment (c).

The actin cytoskeleton, a dynamic filamentous network, contributes to cell shape maintenance, cellular contractility, and motility. It exists in a dynamic equilibrium between monomeric G-actin and polymeric F-actin forming actin fibers. Sex differences in actin filament morphology were detectable in CDCs at basal conditions (Figure 2(a)). Numerous ventral stress fibers were clearly visible by static cytometry, that is, fluorescence microscopy, in cells from males whereas transverse arcs were detected in cells from females. Ventral stress fibers generate strong traction forces at the cell base. They lie along the base of the cell, being usually parallel to the direction of migration, and they are attached to the focal adhesions at both ends [38]. Transverse arcs, instead, are curved stress fibers found parallel to the leading edge and are assembled from shorter actin filaments that originate in the lamellipodium. They are contractile and do not attach to focal adhesions [38]. Interestingly, short exposure (3 h) to TNF-*α* at low concentration induced a clear redistribution of actin filaments, including stress fibers loss and intense lamellipodia formation in both male and female cells (Figure 2(b)). After a longer exposure time (24 h) to TNF-*α*, these peripheral structures progressively disappeared, whereas stress fibers reappeared (Figure 2(c)). We cannot rule out the possibility that differences in cytoskeletal network between cells from males and females could modulate the cytoplasmic trafficking of molecules such as NF-*κ*B [39].

### 3.3. TNF-*α* Influences Cardiac Progenitor Cell Migration Ability

**Figure 3:**
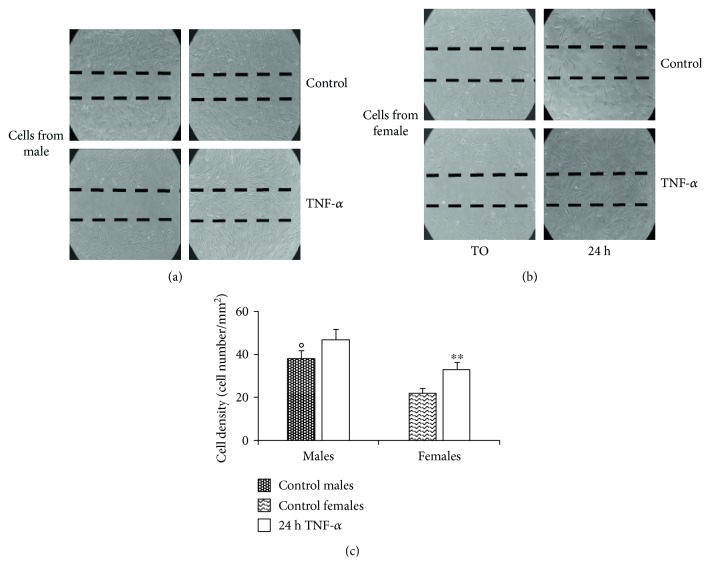
TNF-*α* influences cardiac progenitor cell migration ability. Migration test performed by scratch assay on CDCs from (a) male and (b) female patients. Images were captured by phase-contrast microscopy using a 4.6x objective at 0 and 24 hours after TNF-*α* treatment. A representative experiment among three is shown. (c) Histogram showing cell density (cell number/mm^2^). A significant difference was found between cells from males and females (°*p* < 0.05). TNF-*α* promotes significant (^∗∗^*p* < 0.001) cell migration only in CDCs from female patients. The results represent the mean ± SD of three independent experiments.

CPCs' ability to migrate towards a damaged site plays an important role in their regenerative response. To this regard, 24 hours after the scratch on confluent CDC cultures, a significant difference was found between cells from males and females (*p* < 0.05; 36 cells/mm^2^ for males and 21 cells/mm^2^ for females) (Figures 3(a) and 3(b)). In particular, treatment with TNF-*α* at low concentration promoted migration of CDCs. However, this “booster” effect was significant (*p* = 0.001) only for cells from female patients (Figure 3(c)).

## 4. Conclusions

Modern surgical and medical therapies have been proven to be still unsatisfactory in the optimal prevention and treatment of heart failure. Moreover, in the perspective of personalized medicine, significant insights are gradually suggesting differential pathophysiological responses based on the patient's sex [40, 41]. This is particularly true for cardiovascular diseases [3, 42] and cardiac regenerative medicine [43], which may ultimately mirror, at least in part, a different potential and/or responsiveness of resident S/PC pools in terms of function and repair. Genetic (e.g., sex chromosomes), epigenetic (e.g., microRNA), or metabolic (e.g., redox regulation) mechanisms should be taken into consideration in order to explain differences between cells from males and females [44–47].

In the present work, we investigated in vitro the differential sex-related biological response of human resident CPCs in the form of CDCs to TNF-*α*, an inflammatory cytokine implicated in ischemia/reperfusion, whose effects are bidirectional and dependent on the activation of its receptors and on cytokine levels. TNFR1 activation generates ROS and induces apoptosis, while TNFR2 activation leads to cell survival, proliferation, and growth factor production [19, 48]. In our work, we found that, although both TNFR1 and TNFR2 are expressed by male and female CDCs, the intracellular signaling mostly activated in our experimental conditions is apparently due to TNFR2, since cell cycle progression and cell spreading seem to be improved, whereas cell death remains negligible at the TNF-*α* concentration considered here. More importantly, we also found that the response to TNF-*α* significantly differed between CDCs from females and males. In particular, CDCs from females appear more responsive to TNF-*α* treatment in terms of cell cycle progression and cell migration ability. Our results are in line with reported data, such as greater myocardial protection capacities exerted by female versus male MSCs [43] or paracrine potentiation of MSCs and CPCs by estradiol treatments [49, 50]. Considering the differences in the incidence of cardiovascular diseases in men versus women, these results suggest that cell therapy protocols should take into account the donor sex in order to improve their efficacy. Allogeneic CDC transplantation has been shown to combine beneficial therapeutic effects with negligible effects due to immune issues [17, 51].

Hence, in view of difficulties encountered in the development of this area of research (e.g., difficulties in S/PCs culture, as well as in transplantation protocols and engraftment efficiency), the present report supports the conclusion that TNF-*α*-stimulated CDCs isolated from heart biopsies of female donors could represent an improved and promising candidate cell population for cardiac regenerative therapy applications.

## Supplementary Material

Supplementary figure 1. Gene expression levels for a selected panel of cardiovascular and mesenchymal markers (A) and for NADPH-oxidase (NOX) isoforms (B) in cardiosphere-derived cells (CDCs) from female or male donors (n=6 each). Data is plotted as 2^-ΔCt, using GAPDH as the housekeeping gene, and presented as mean +/− standard error of the mean.

## Figures and Tables

**Table 1:** Main available morphometric and medical characteristics of donor patients.

	Sex	Age	BMI	Diagnosis	Diabetes	Smoke
(1)	F	79	27,7	IC	N	N
(2)	M	58	22	IC	Y	Y
(3)	M	64	27,4	MI	N	Y
(4)	F	72	32,5	IC, AS, MI	Y	N
(5)	M	58	28,4	IC	Y	Y
(6)	F	63	32,9	IC, AS	Y	N
(7)	M	73	28	IC	N	N
(8)	M	54	30	IC	N	Y
(9)	F	78	21	IC, AD	N	Y

BMI: body mass index; IC: ischaemic cardiomyopathy; AS: aortic stenosis; MI: mitral insufficiency; AD: aortic dissection.
