# Supplementary material for: Sex Differences of Human Cardiac Progenitor Cells in the Biological Response to TNF-α Treatment
Source: Stem Cells Int. 2017 Sep 17;2017:4790563. doi: 10.1155/2017/4790563 (PMC5623773; doi:10.1155/2017/4790563)

**SUPPLEMENTARY MATERIAL**

**Sex differences of human cardiac progenitor cells in the biological response to TNF-α treatment**

Elisabetta Straface*^1^, Lucrezia Gambardella^1^, Francesca Pagano^2^, Francesco Angelini^2^, Barbara Ascione^1^, Rosa Vona^1^, Elena De Falco^2^, Elena Cavarretta^2^, Raffaele La Russa^3,4^, Walter Malorni^1#^, Giacomo Frati^2,5#^, Isotta Chimenti^2#^.

^1^Center for Gender-Specific Medicine, Istituto Superiore di Sanità, Italy

^2^Department of Medical Surgical Sciences and Biotechnologies, “La Sapienza” University of Rome, Italy.

^3^Department of Anatomical, Histological, Forensic and Orthopaedic Sciences, Sapienza University of Rome, Viale Regina Elena 336, 00185 Rome, Italy;

^4^Malzoni Clinical-Scientific Institute (MaCSI), Via Carmelo Errico 2, 83100 Avellino, Italy;

^5^Department of AngioCardioNeurology, IRCCS Neuromed, Pozzilli, Italy.

^#^These authors equally contributed.

**Supplementary figure 1.** Gene expression levels for a selected panel of cardiovascular and mesenchymal markers (A) and for NADPH-oxidase (NOX) isoforms (B) in cardiosphere-derived cells (CDCs) from female or male donors (n=6 each). Data is plotted as 2^-ΔCt, using GAPDH as the housekeeping gene, and presented as mean +/- standard error of the mean.


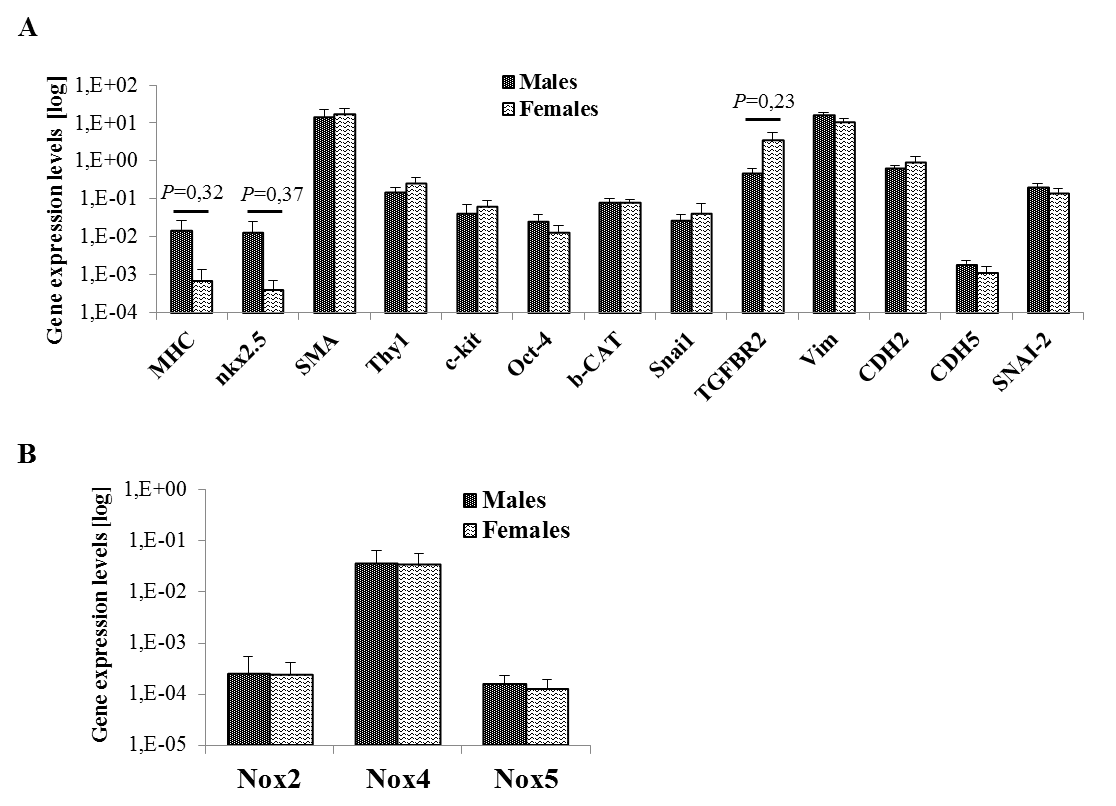

Supplement: Supplementary file 1 — Supplementary figure 1. Gene expression levels for a selected panel of cardiovascular and mesenchymal markers (A) and for NADPH-oxidase (NOX) isoforms (B) in cardiosphere-derived cells (CDCs) from female or male donors (n=6 each). Data is plotted as 2^-ΔCt, using GAPDH as the housekeeping gene, and presented as mean +/− standard error of the mean. [file 4790563.f1.docx]
